# Wide-awake local anaesthesia no tourniquet-assisted subcutaneous anterior ulnar nerve transposition in cubital tunnel syndrome: A retrospective evaluation

**DOI:** 10.1097/MD.0000000000043921

**Published:** 2025-08-08

**Authors:** Ümit Gök, Ferhat Öktem, Çağdaş Pamuk, Muhammed Fatih Serttaş, Hüseyin Olgun, Seyit Ali Güçlü

**Affiliations:** a Orthopedics and Traumatology Department, Kocaeli City Hospital, University of Health Science, Kocaeli, Turkey; b Orthopedics and Traumatology Department, Private Silivri Anadolu Hospital, İstanbul, Turkey.

**Keywords:** cubital tunnel syndrome, entrapment neuropathy, WALANT

## Abstract

Although several studies have demonstrated the successful use of wide-awake local anesthesia no tourniquet (WALANT) technique in cubital tunnel decompression, no prior study has investigated its use in anterior ulnar nerve transposition. This study aimed to retrospectively evaluate the outcomes of patients who underwent ulnar nerve decompression and subcutaneous anterior transposition using WALANT. A total of 41 surgeries were performed on 40 patients between August 2012 and March 2024 by a single surgeon. Patient data including age, sex, surgical side, Dellon satisfaction scale scores, postoperative complications, and satisfaction with anesthesia were collected. All procedures were performed using WALANT. Twelve (30%) patients were female and 28 (70%) were male and the mean postoperative follow-up period was 5.8 weeks (range 4–9 weeks). In the preoperative evaluation according to the Dellon scale, 12 (30%) patients had mild disease, 23 (57.5%) had moderate moderate, 5 (12.5%) had severe disease. Patient satisfaction was evaluated as bad in 3 patients (7.5%), moderate in 11 patients (27.5%) and good in 26 patients (65%). A significant negative correlation was observed between age and satisfaction questionnaire scores (*r* = −0.27, *P* = .023). Age also had a significant effect on the likelihood of conversion to general anesthesia (*P* = .0018). The WALANT anesthesia technique for anterior subcutaneous transposition of the ulnar nerve in cubital tunnel syndrome is safe and effective. The most important point in this regard is patient selection. A prospective cohort study with a larger cohort and longer follow-up time is required to monitor the long-term clinical outcomes.

## 1. Introduction

Ulnar nerve entrapment at the level of the cubital tunnel is the second most common nerve entrapment syndrome after the carpal tunnel and surgical treatment is usually performed under general or regional anesthesia.^[1]^ Tourniquets are commonly used during surgery. In simple cubital tunnel syndrome (CuTS), release of the ulnar nerve in the cubital tunnel is sufficient; however, in more complex cases, additional interventions such as transposition of the ulnar nerve or medial epicondylectomy may be required.^[1,2]^

Wide-awake local anesthesia no tourniquet (WALANT) has gained popularity, especially after the COVID-19 pandemic, and is spreading rapidly, especially among hand surgeons. The advantages of this technique include being more minimally invasive than general and regional anesthesia, not requiring patient compliance with anesthesia as in general anesthesia, providing preoperative motor function control, and not requiring inpatient follow-up for anesthesia. In addition, by adding epinephrine to the local anesthetic, the amount of bleeding was reduced and better visualization was provided. However, the patient should be calm and relaxed during surgery and should be able to comply with verbal commands.^[3,4]^

In related studies, it has been shown that successful results have been obtained in surgical decompression of the ulnar nerve in the cubital tunnel with the WALANT, but no study on anterior transposition of the ulnar nerve was found in the literature. Even in Bruggink study, patients requiring anterior transposition were excluded.^[1]^ In this study, we hypothesized that anterior nerve transposition during ulnar nerve decompression surgery under WALANT is possible and may have a positive effect on functional outcomes.

This study aimed to retrospectively evaluate patients who underwent decompression and anterior subcutaneous transposition of the ulnar nerve in the cubital tunnel using the WALANT.

## 2. Methods

In this cross-sectional study, the records of patients with a diagnosis of cubital tunnel syndrome (ICD code G56.2) who underwent ulnar nerve decompression and anterior transposition surgery performed by the same surgeon between August 2012 and March 2024 in the Orthopedics and Traumatology Department of İzmit SEKA State Hospital and Kocaeli City Hospital were retrospectively evaluated. Patient data were collected from the patients’ files and contacted by phone. Prior to the study, approval was obtained from the Kocaeli City Hospital Scientific Research Ethics Committee on 27.02.2025, with protocol code 2025.18. Written informed consent was obtained from all patients. This study adhered to the Declaration of Helsinki standards revised in 2013.

### 2.1. Patients

Between August 2012 and March 2024, 46 surgeries (1 patient bilaterally) of 45 patients underwent ulnar nerve decompression and ulnar nerve anterior transposition surgery.

#### 2.1.1. Inclusion criteria

All patients were diagnosed with cubital tunnel syndrome supported by electromyography (EMG), which did not improve despite conservative treatment (rest, nonsteroidal anti-inflammatory drug [NSAID] therapy, physical therapy, and splinting).

CuTS was diagnosed based on clinical findings and was later confirmed using electrophysiological tests. Clinical findings include sensory impairment of the ulnar nerve sensory region and motor dysfunction triggered by intrinsic muscle atrophy. Surgical treatment was performed in patients who still had significant symptoms of tingling, pain, or weakness after at least 2 months of conservative therapy and 2 steroid injections (40 mg methylprednisolone acetate [Depo-Medrol; Pfizer, New York] and 10 mg lidocaine hydrochloride). Before injection, the medial epicondyle and course of the ulnar nerve were marked with a surgical marker, and all steroid injections were performed with the elbow at 90° flexion under local anesthesia using a noncontact technique with topical vapocoolant spray just posterior to the medial epicondyle.

#### 2.1.2. Exclusion criteria

Patients who underwent decompression alone, neurologically unstable patients and patients under 18 years of age were excluded from the study.

One neurologically unstable patient, 2 patients under 18 years of age, and 2 patients whore fused to provide written consent were excluded from the study.

### 2.2. The examined variables

Age, sex, side, comorbidities, postoperative complications, visual analog score and satisfaction with anesthesia were recorded. Clinically, the patients were classified according to the Dellon Symptom Severity Scale as: mild, moderate, or severe (Table 1).

**Table 1:** Characteristics and postoperative results of patients (EMG, VAS).

Age	43.56 (19–81)
Sex	
Female	12 (30%)
Male	28 (70%)
Side	
Right	15 (15.75%)
Left	24 (60%)
Bilateral	1 (2.5%)
Smoking	17 (42.5%)
Alcohol	3 (7.5%)
Diabetes mellitus	6 (15%)
Hypertension	4 (10%)
Hyperthyroidism	1 (2.5%)
Hypothyroidism	1 (2.5%)
Preoperative symptoms	
Mild	12 (30%)
Moderate	23 (57.5%)
Severe	5 (12.5%)
Positive EMG	40 (100%)
Preoperative satisfaction	
Good	26 (65%)
Mild	11 (27.5%)
Poor	3 (7.5%)
VAS score	
Preoperative	7.76
Postoperative	1.45 (*P* = .007)
Complications (early)	
Hematoma	4 (10%)
Paresthesia	9 (22.5%)
Pain	3 (7.5%)
Infection	0 (0%)
Up to 6th mo	
Paresthesia	4 (10%)
Pain	1 (2.5%)
Up to 1st yr	
Paresthesia	3 (7.5%)

EMG = electromyography, VAS = visual analog score.

#### 2.2.1. Dellon symptom severity scale

Mild

Sensory: intermittent paresthesia vibratory perception increased
Motor: subjective weakness, clumsiness, or loss of coordination
Tests: elbow flexion test, Tinel sign, or both are positive

Moderate

Sensory: intermittent paresthesia vibratory perception normal or decreased
Motor: measurable weakness in pinch or grip strength
Tests: the elbow flexion test, Tinel sign, or both positive finger crossings may be abnormal

Severe

Sensory: paresthesia are persistent vibratory perception decrease abnormal 2 point discrimination
Motor: measurable weakness in pinch or grip strength plus muscle atrophy
Tests: the elbow flexion test, Tinel sign, or both are positive Finger crossing usually abnormal

#### 2.2.2. Satisfaction

During the postoperative period, patients were asked to score their level of satisfaction with the surgical procedure, between 0 and 10 (0; completely dissatisfied, 10; maximum satisfaction). The satisfaction questions included fear of anesthesia, fear of surgery, waiting time for anesthesia, and waiting for a perioperative position. Considering these scores, satisfaction was grouped as follows;

Good: satisfaction score > 7,
Moderate: satisfaction score 4 or 5,
Poor: satisfaction score < 4

#### 2.2.3. Intraoperative pain

In postoperative period, patients were asked to score their level of intraoperative pain with the surgical procedure, between 0 and 10 (0: no pain, 10: maximum pain). Based on these scores, pain was grouped as follows:

Good: pain score < 4,
Moderate: pain score 4 or 5,
Poor: pain score > 7

### 2.3. Surgical procedures

**Figure 1.**
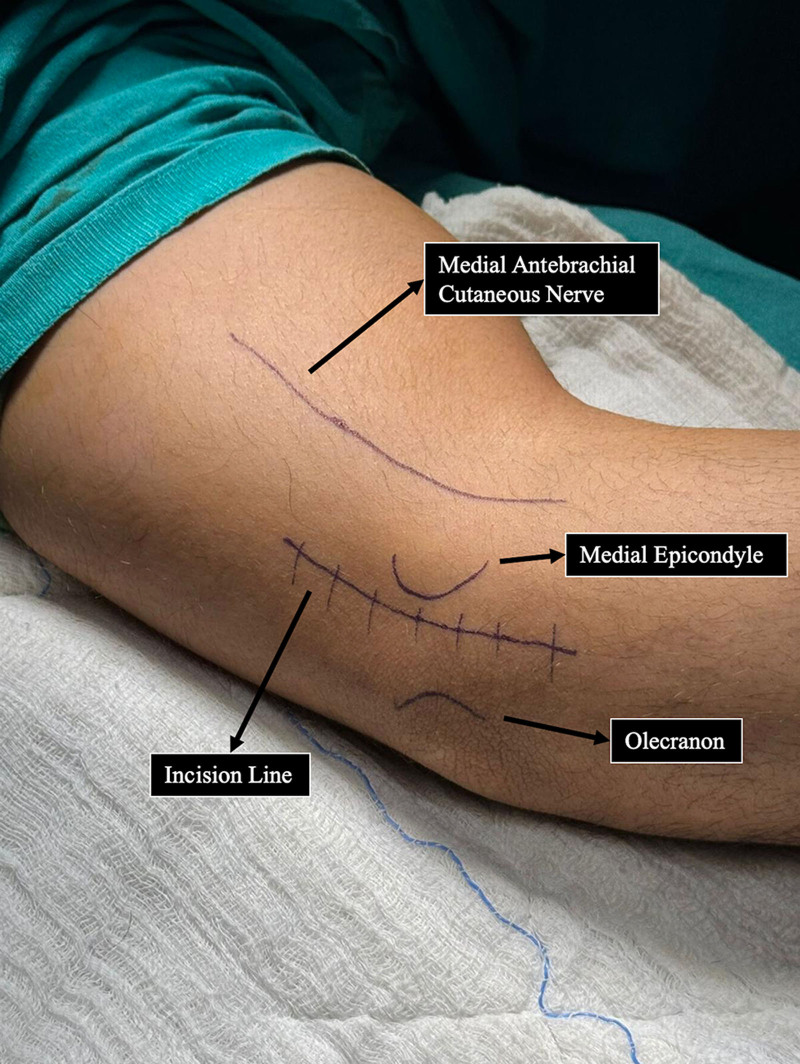
Anatomic landmarks of surgical procedure.

**Figure 2.**
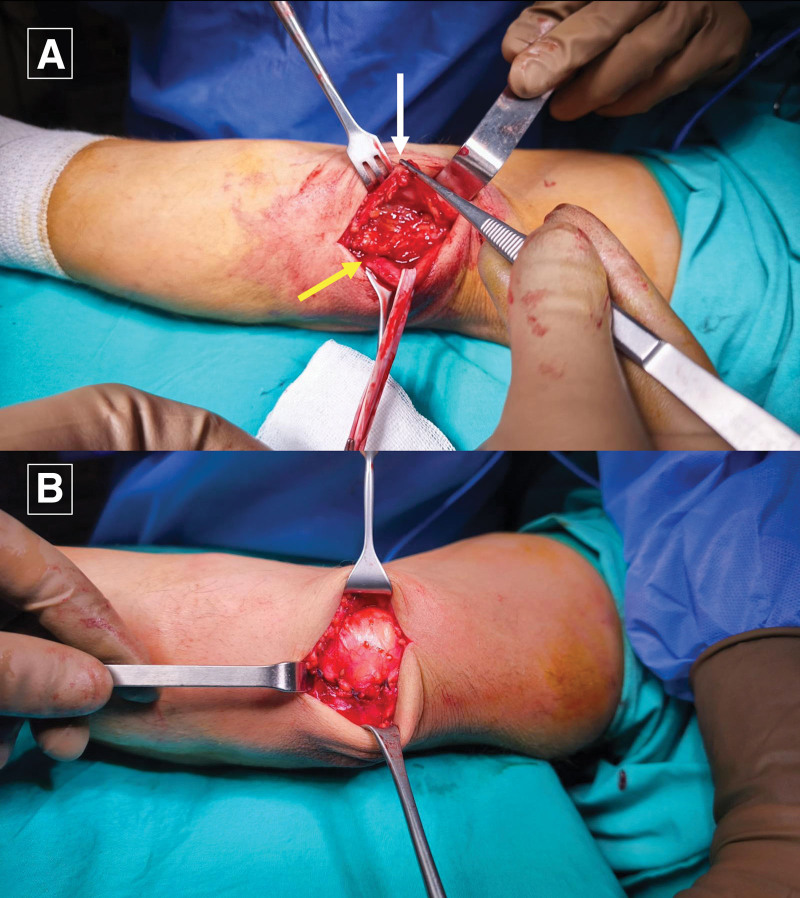
(A) Ulnar nerve release (B) Closure after anterior subcutaneous transposition of ulnar nerve.

All the patients underwent surgery under WALANT anesthesia. The local anesthetic consisted of 1% lidocaine with 1:100,000 epinephrine and was buffered with 8.4% bicarbonate in a 10:1 ratio.^[4]^ It was administered to the medial antebrachial cutaneous nerve area and incision line (Fig. 1). The patient was then requested to wait for approximately 30 minutes before starting surgery. After decompression of the ulnar nerve, we evaluated nerve stability in full flexion and performed subcutaneous anterior ulnar nerve transposition (Fig. 2). Before transposition, 5 cc of prilocain was applied to the anterior subcutaneous layer and left for 5 minutes. The subcutaneous layer and skin were closed and the wound was dressed. After the surgery the patient received a compressive bandage and was hospitalized overnight. Active use of the entire arm was permitted immediately after the third day of surgery, but heavy lifting and intensive use of the arm were permitted only after 4 weeks.

Postoperatively, follow-up visits were performed at 2 weeks, 6 weeks, 6 months, and annually, thereafter. All patients were followed for at least 1 year. All clinical assessments were performed by a single investigator blinded to the surgical procedure. Data including the duration of symptoms, occupation of the patients, Tinel sign over the course of the ulnar nerve in the postcondylar groove, Froment and Wartenberg signs, elbow flexion test, and palpation for any subluxation of the nerve were recorded pre and postoperatively. Grip and key-pinch strengths were also evaluated (Jamar dynamometer; Preston, Jackson, MI, USA) by the same investigator preoperatively and at the final follow-up visit (Table 2). Additionally, postoperative complications were noted and patient satisfaction was evaluated based on the status of the patients’ return to their former job/daily activities.

**Table 2:** Neurological findings for study groups and statistical comparison results.

	Preoperative (n) (%)	Postoperative (n) (%)	*P*-value
Tinel sign	35	3	.001
Elbow flexion test	28	0	.001
Froment sign	12	1	.012
Wartenberg sign	6	1	.008
Nerve subluxation	2	0	.076
Grip strength (%)	78	90	.024
Key-pinch strength (%)	76	91	.018

### 2.4. Statistical analysis

Statistical analyses were performed using IBM SPSS 29.0 for Windows version 2.0 software (IBM, Armonk) and the statistical significance level was accepted as *P* < .05. The chi-square test was used for nominal variables (sex, preoperative anesthesia change, etc), Spearman correlation analysis was used for the relationship between continuous variables (age, body mass index, satisfaction questionnaire, postoperative elbow stiffness, etc), and the Mann–Whitney *U* test was used for the relationship between nominal and continuous variables. Descriptive data were expressed as mean ± standard deviation, median (min–max), or number and frequency.

## 3. Results

Forty patients were retrospectively screened and found suitable for this study. The patients’ demographic characteristics are shown in Table 1.

Twelve (30%) patients were female and 28 (70%) were male, and the mean postoperative follow-up period was 5.8 weeks (range 4–9 weeks). 24 (60%) patients underwent surgery on the left side, 15 (37.5%) on the right side and 1 (2.5%) on both sides (in different sessions). The mean age was 43.56 years (range 19–81) and all patients had positive EMG findings. In preoperative evaluation According to the Dellon scale, 12 (30%) patients had mild disease, 23 (57.5%) had moderate disease, 5 (12.5%) had severe disease. Seven (17.5%) patients underwent surgery under general anesthesia because they could not comply during surgery. Hematoma developed in 4 (10%) patients in the postoperative period, which resolved spontaneously in the second week. No postoperative infection was observed in any of the patients. Postoperative pain and elbow movement limitations due to hematoma resolved in all patients by the ninth week. In the postoperative period, pain due to ulnar neuropathy persisted in 1 (2.5%) and parasthesia in 4 (10%) patients until the 6th month. At the end of the first year, paresthesia persisted in 3 (7.5%) patients.

Patients’ satisfaction with perioperative anesthesia and surgery was evaluated subjectively as good, fair or poor. Surgery using the WALANT technique was evaluated as bad in 3 patients (7.5%), moderate in 11 patients (27.5%) and good in 26 patients (65%). The reason for the poor evaluation was the difficulty experienced during surgery in 3 patients. The perioperative satisfaction of the patients who continued general anesthesia due to pain was good.

There was a statistically significant negative correlation between age and satisfaction questionnaire scores (*r* = −0.27, *P* = .023). This finding suggests that satisfaction levels may decrease with age.

In the statistical evaluation between age and perioperative anesthesia change, it was found that age had a statistically significant effect on anesthesia change (*P* = .0018) and the need for preoperative general anesthesia decreased as age increased.

There was no significant correlation between postoperative elbow stiffness and age, sex, perioperative satisfaction status, or body mass index (Table 3).

**Table 3:** Statistical analysis of data (BMI).

Comparison	*P*-value
Gender versus preoperative satisfaction	.569
Gender versus preoperative anesthesia change	.342
Age versus preoperative satisfaction	.023
Age versus preoperative anesthesia change	.0018
BMI versus preoperative satisfaction	.575
Preoperative anesthesia change versus preoperative satisfaction	.214
Postoperative stiffness versus age	.958
Postoperative stiffness versus gender	.675
Postoperative stiffness versus preoperative satisfaction	.831
Postoperative stiffness versus BMI	.114

BMI = body mass index.

## 4. Discussion

In this study, it was observed that the WALANT can be used safely in the surgical treatment of ulnar nerve entrapment neuropathy; anterior transposition of the ulnar nerve under the WALANT is possible and high patient satisfaction scores can be obtained.

Although there is no consensus on which method should be chosen for the treatment of cubital tunnel syndrome, decompression of the ulnar nerve in the cubital tunnel, medial epicondylectomy and endoscopic ulnar decompression have been reported in literature.^[1,2,5]^ Surgeons who prefer anterior subcutaneous transposition claim to experience less pain and less tension on the nerve in the postoperative period owing to early mobilization. Surgical treatment is usually performed under general or regional anesthesia.^[1]^ WALANT has become increasingly popular among orthopedists. However, its use, especially in the elbow region, is more limited than that in the hand and wrist. There are also studies comparing general anesthesia with the WALANT technique in ulnar nerve decompression but no differences were observed.^[5,6]^ However, Bruggink et al stated that the WALANT technique is contraindicated in patients requiring anterior transposition of the ulnar nerve.^[1]^ We showed that good and satisfactory results can be obtained with the WALANT technique for anterior transposition of the ulnar nerve after decompression. The fact that 1 patient in our study wanted to undergo surgery on the other side with the same technique after the first-side surgery is a good case supporting our findings.

Successful surgical procedures performed with WALANT have been reported in the literature in both patient groups aged 18 and over 65 years.^[7,8]^ In our study, we found a significant correlation among age, perioperative anesthesia changes, and perioperative satisfaction scores. In older patients, this anesthesia change was not observed; however, the satisfaction score was significantly poorer in the older group. We thought that the younger patients had more anxiety and the older age group (especially over 60 years old) had higher comfort expectations. Similar to the literature, we believe that better results can be achieved in all age groups if preoperative and perioperative patient communication and information about the procedure to be performed are better and more detailed.^[9–11]^

Although the same surgical procedures are applied, the advanced stage of the disease and increased nerve damage prolong the time required for complaints to decrease or disappear completely in the postoperative period. Matsuzaki et al showed that patients with severe intrinsic muscle atrophy and inadequate motor and sensory nerve conduction velocities can expect satisfactory long-term functional results after surgery; however this process takes longer in the elderly. They also showed that function and sensory function continue to improve after 2 years and that the process is more advanced in the elderly.^[12]^ Bruggink et al showed that paresthesia after ulnar decompression under the WALANT procedure persisted in 10 patients 3 months postoperatively.^[1]^ In our study, paresthesia persisted after the 6th month in 4 patients and in 3 of them paresthesia persisted at the end of the 1st year. We think that this may be due to the fact that the follow-up period in these patients was limited to 1 year. However, nerve palsy and muscular hypotrophy present preoperatively did not increase postoperatively in any of the patients.

An adequate explanation of the entire procedure using the WALANT technique is necessary for patients undergoing surgery. Surgeons should evaluate the patient’s personality before the WALANT technique, as patients with psychological problems or excessive anxiety are contraindications to the WALANT procedure. It is recommended that patients who are not suitable for this technique undergo surgery under general anesthesia.^[13]^ Although cognitive impairment was assessed preoperatively, dissatisfaction due to perioperative anxiety was observed in 3 patients in our study. In addition, although 7 patients were converted to general anesthesia preoperatively, we believe that increased anxiety was effective in these 7 patients because they had good satisfaction with the procedure. Therefore, we believe that a more careful psychological evaluation should be performed during preoperative patient selection.

The use of local anesthesia is risky. Risk factors for the systemic toxicity of local anesthesia include age, hepatic dysfunction, low cardiac output, cardiac pathology, pregnancy, and extreme use of β-blockers, digoxin, and calcium antagonists.^[14]^ Lidocaine with epinephrine can be cardiotoxic; however lidocaine anaphylaxis is rare. Epinephrine in lidocaine may cause a transient increase in the heart rate and blood pressure however, its clinical significance remains uncertain.^[14]^ No complications related to the use of local anesthetics or epinephrine were observed in our study.

Previous studies, have reported that surgical procedures such as trigger finger release, which are performed in smaller areas, can be performed in procedure rooms under field sterility with very low infection rates.^[15–18]^ However, in surgeries with larger and deeper incisions, such as the surgical treatment of distal radius fractures using the WALANT procedure, general operating room conditions are recommended.^[12]^ In our hospital, anterior ulnar transfer using the WALANT technique is still performed in the main operating room. Preoperative intravenous antibiotics containing 1 g cefazolin were administered to each patient as prophylaxis to prevent infection. Despite the prolonged surgical time due to anterior transfer, no infection was observed in our study.

Although studies on ulnar decompression have reported postoperative local infection, muscle hypotrophy, prolonged hematoma, and elbow stiffness, these complications were not observed in our study.^[5,10]^ However, the hematoma that occurred in 4 patients did not cause any late complications in the elbow. In particular, although the amounts of bleeding reported in these studies was very low, it has been reported that these small amounts of bleeding can also cause problems because the study field is narrow.^[7,8,12,13]^ We believe that well-performed perioperative hemostasis is sufficient for safe bleeding control, although additional doses of adrenaline are not required for ulnar nerve release or anterior transposition.

The main limitations of our study are that it was retrospective and the number of cases was not very high. On the other hand, the sex distribution and socioeconomic status of the patients showed an almost homogeneous distribution. In addition, we consider that all operations were performed in the same hospital and by the same surgeon, which is one of the strengths of the present study.

## 5. Conclusion

The WALANT anesthesia technique for anterior subcutaneous transposition of the ulnar nerve in cubital tunnel syndrome is safe and effective. The most important point in this regard is compatible patient selection. A prospective cohort study with a larger cohort and longer follow-up time is required to monitor the long-term clinical outcomes.

## Author contributions

**Conceptualization:** Hüseyin Olgun.

**Data curation:** Ümit Gök, Çağdaş Pamuk, Hüseyin Olgun, Seyit Ali Güçlü.

**Formal analysis:** Çağdaş Pamuk, Seyit Ali Güçlü.

**Investigation:** Muhammed Fatih Serttaş.

**Methodology:** Ümit Gök, Ferhat Öktem.

**Writing – original draft:** Ümit Gök, Çağdaş Pamuk.

**Writing – review & editing:** Muhammed Fatih Serttaş.
